# Smart probe for simultaneous detection of copper ion, pyrophosphate, and alkaline phosphatase in vitro and in clinical samples

**DOI:** 10.1007/s00216-019-02027-2

**Published:** 2019-08-02

**Authors:** Sonia Kiran, Renuka Khatik, Romana Schirhagl

**Affiliations:** 1 Department of Chemistry, University of Science and Technology of China, #96 Jinzhai Road, Hefei, 230026 Anhui China; 2 University Medical Center Groningen, Groningen University, Antonius Deusinglaan 1, 9713 AW Groningen, The Netherlands

**Keywords:** Wilson’s disease, Copper ion, Pyrophosphate, Alkaline phosphatase, Fluorescent probe

## Abstract

Wilson’s disease (WD), which might lead to acute liver failure, is an inherited disorder characterized by accumulation of copper (Cu^2+^) in the brain, the liver, and other vital organs. In the clinic, decreased serum alkaline phosphatase (ALP) concentration is used for WD diagnosis. But to the best of our knowledge, using a fluorescent probe to simultaneously detect multiple factors in WD (e.g., Cu^2+^, pyrophosphate (PPi), and ALP) has not been reported. Herein, we rationally designed a fluorescent switch (E)-8-((4-methylbenzylidene)amino)napthalen-1-amine (**L**) and successfully applied it for sequential and selective detections of Cu^2+^, PPi, and ALP in vitro, in living cells and synovial fluid samples with “Off,” “On,” and “Off” fluorescence signals, respectively. Considering the obvious correlations among Cu^2+^, PPi, and ALP in WD, we envision that our fluorescent probe **L** could be applied to in vitro diagnosing WD in the near future.

**Electronic supplementary material:**

The online version of this article (10.1007/s00216-019-02027-2) contains supplementary material, which is available to authorized users.

## Introduction

Copper is present as trace element in the human body. It is crucial for various physiological activities like functioning of proteins, expressing genes [1], and operating of the human nerve system [2]. Dearth of copper ion (Cu^2+^) results in myelopathy [3] and exalted level of Cu^2+^ may lead to kidney and liver damage [4]. For sensitive discernment of Cu^2+^, different techniques such as surface plasmon resonance (SPR) [5], atomic absorption/emission spectroscopy (AAS/AES) [6], inductively coupled plasma mass spectroscopy (ICPMS) [7], and surface-enhanced Raman scattering (SERS) [8] have been successfully applied hitherto. However, costly equipment and sophisticated and well-trained personnel are required for these approaches.

Small molecule–based (SMB) systems are potentially able to overcome the obstacles. For examples, nucleic acid sensors [9] and new “Off” (or “On”) fluorescent probes [10–14] have been introduced for the detection of Cu^2+^ with excellent sensitivity and good accuracy. However, these systems also have some snags. Often they require harsh reaction conditions, tedious sample preparation procedures, and chemical and physical interference with coexisting metal ion or inadequate biocompatibility [15].

Beside cations, anions are also essential in biological processes and industrial units and have gained attention in the last decades [16]. One biologically significant anion is pyrophosphate (PPi) which is product of adenosine triphosphate (ATP) metabolism [17]. PPi concentration provides indispensible information about replication of DNA [18]. The eminent level of PPi in the synovial fluid has been suggested as a disease marker in arthritis patients; hence, PPi serves as biomarker for calcium pyrophosphate dihydrate (CPPD) crystal deposition or chondrocalcinosis [19, 20]. Until now, various techniques had been used for PPi detection, such as colorimetric assays [21], electrooptical methods [22], and fluorescence assays [23]. Advantages of fluorescent chemosensors are their low cost, high spatial resolution and sensitivity, and short response time [24]. The strong binding interaction between metal ion and PPi opens different ways to design metal ion complex–based fluorescent switch as useful approach for PPi detection [25].

Alkaline phosphatase (ALP) is an indispensible enzyme which catalyzes the dephosphorylation of proteins, small molecules, and nucleic acids [26]. Deviated ALP level might induce various disorders such as diabetes, bone diseases, hepatobiliary disease, breast cancer, and prostatic cancer [27]. Hence, it is decisive to establish sensitive and simple methods for accurate detection of ALP level. Up to date, many different approaches have been applied to recognize ALP, such as electrochemical assays [28], fluorescence [29], chemiluminescence [30], SERS [31], and capillary electrophoresis [32]. The fluorescent assays of ALP have captivated much consideration due to their reliability, accessibility, and sensitivity [33]. Su et al. developed a carbon quantum dot–based fluorescent switch for ALP sensing [34–36]. Xiang and coworkers reported a fluorescent sensor for ALP quantification utilizing bright fluorescent nanosheets of g-C_3_N_4_ [28]. Liang et al. utilize sol-gel transition (hydrogelation) with “Turn-Off” fluorescence for quantitative analysis of ALP both in vitro and in LoVo cells [37].

Wilson’s disease (WD) is an inherited disorder characterized by an excess of copper in the liver and brain. Severe WD might lead to acute liver failure which is often fatal. In genomics, WD is induced by the mutation in the Atp7b gene which codes the copper-transporting P-type ATPase involved in cellular copper excretion [38]. Patients suffering from Wilson’s disease exhibit high urinary copper levels (> 100 mg/day, compared with 20–40 mg/day in healthy individuals) and increased serum-free copper levels (> 25 μg/dL, compared with 11–25 μg/dL in healthy individuals) [38]. As a result, decreased serum ALP concentration (the ratio between serum ALP to bilirubin is < 2) and non-immune hemolytic anemia are often seen in WD and the former is used for WD diagnosis in the clinic [39].

Considering the relationships among Cu^2+^, PPi, and ALP in WD, we designed a fluorescent switch for the sequential and selective detections of these three analytes. Such a combined detection scheme has already developed before making use of different kinds of nanoparticles. More specifically, Mo oxide quantum dots [40], carbon dots [41–43], Au nanoclusters [44], carbon dots and Au nanoclusters [45], silver nanocluster [46], upconversion nanoparticle [47], Eu(DPA)3@Lap nanohybrid material [48], or anionic conjugated polymers [49] were utilized. Several of these have superior sensitivity. However, these are comparably large and thus sensing resolution is limited. Also most of them contain heavy metals which can be a biocompatibility concern. The existing literature is compared with this work in Table 1.

**Table 1:** comparison of combined essays (for more comparisons with single component sensors, see Electronic Supplementary Material (ESM))

Ref	LOD	Linear range	Response time	Sample
40	0.02 U/L	0.1–5 U/L	10 min	Human serum
41	NA	NA	10 min	Cell cultures
42	0.1 U/L	0.1–75 U/L	Minutes	Chemical samples
43	0.05 U/L	0.12–15 U/L	Minutes	Mineral water
44	0.000005 U/L	0.03–3 U/mL	30 min	Clinical samples
45	0.019 U/mL	0.0625–0.875 U/mL	10 min	Diluted bovine serum/cells
46	0.5 U/L	0.5 to 60 U/L	60 min	Chemical samples
47	20 nM	20–100 nM	10 min	Cells
This work	0.4 U/mL	0.4–3.0 U/mL	30 min	Clinical samples

**Scheme 1:**
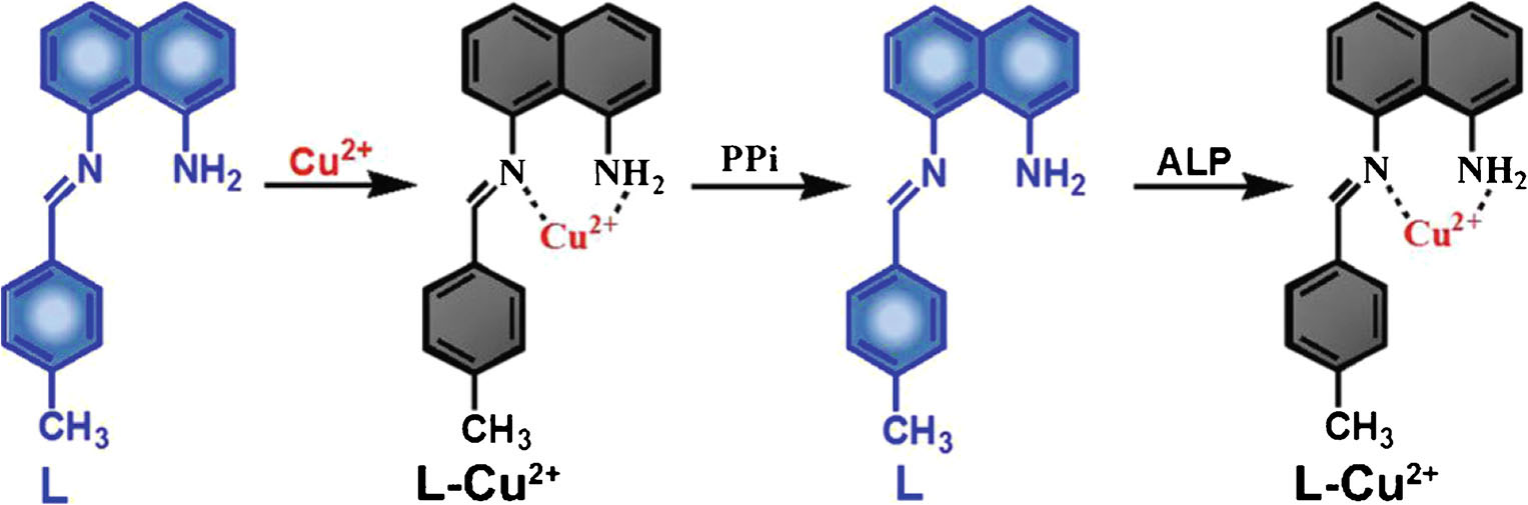
Schematic presentation of fluorescence “Off,” “On,” and “Off” of L for selective detections of Cu^2+^, PPi, and ALP respectively in aqueous buffer and in cells

Here, a fluorescent probe (E)-8-((4-methylbenzylidene)amino)napthalen-1-amine (**L**) which could specifically bind with Cu^2+^ was rationally designed and synthesized (see Scheme 1). Chelation between Cu^2+^ and **L** yields the **L**-Cu^2+^ complex. In this configuration, the fluorescence is “Off.” Due to the stronger binding affinity of PPi-Cu^2+^ than **L**-Cu^2+^, addition of PPi to above solution dissociates the **L**-Cu^2+^ complex and turns the fluorescence “On” again. Interestingly, further added ALP will hydrolyze PPi in the PPi-Cu^2+^ complex, freeing Cu^2+^ to re-chelate with **L** and turn the fluorescence “Off.” With this fluorescence “Off”-“On”-“Off” property, the fluorescence switch **L** was successfully applied for sequential and selective detection of Cu^2+^, PPi, and ALP in vitro and in living cells. A slightly more complex molecule with a similar purpose was reported earlier this year by Pandith et al. [50]. They used a doubly armed hydrazone-based probe (FLRHYDDFP) to continuously detect Cu^2+^, PPi, and ALP with high sensitivity. Compared with Pandith et al., we designed a Schiff base derivative, which has excellent ability for metal ion detection with highly flexible, efficient, selective, and easily applicable structure [51–53]. Our probe was synthesized easily and cheaply by one step. Besides in vitro detection and cell imaging, we also applied our probe for detection of PPi in biological synovial fluid samples. What’s more, we carried out the computational calculations to elaborate the mechanism of fluorescence quenching by ligand to metal charge transfer (LMCT).

## Experimental section

### General methods

#### Materials

Recombinant intestinal ALP was purchased from BaoMan Inc. (Shanghai, China) (one unit is the enzyme activity that cleaves 1 μmol of the standard substrate per minute at 37 °C). The phosphatase inhibitor complex II was purchased from Sangong Biotech Inc. (Shanghai, China) (every 10 μL ALP inhibitor complex II in culture medium containing 1 × 10^7^ cells). All the starting materials were obtained from Sigma or Sangon Biotech. Commercially available reagents were used without further purification, unless noted otherwise.

#### Methods

^1^H NMR and ^13^C NMR spectra were recorded on a Bruker Model AV 300 system. ESI mass spectra were obtained on a Finnigan LCQ Advantage ion trap mass spectrometer (Thermo Fisher Corporation) that was equipped with a standard ESI source, respectively. A F-4600 fluorescence spectrophotometer (Hitachi High-Technologies Corporation, Japan) was used to record fluorescence spectra with excitation wavelengths set to 310 nm. Cell imaging was carried out on a IX71 fluorescence microscope (Olympus, Japan).

### Cell experiments

#### MTT assay

The cervical cancer HeLa cell lines of passage four were kindly provided by Prof Wang, School of Life Science, University of Science and Technology of China. HeLa cells were cultured in Dulbecco’s modified Eagle medium (DMEM), augmented with streptomycin (100 μg/mL) and 10% fetal bovine serum. The cells were cultured in dishes and stored at 37 °C with 5% CO_2_. 3-(4,5-Dimethylthiazol-2-yl)-2,5-diphenyltetrazolium bromide (MTT) assay was used to calculate the cytotoxicity of the probe. HeLa cells were grown into a 96-well cell culture plate for 12 h with 3 × 10^3^ cells/well concentration in humid atmosphere. The solutions of **L** in 100 μL DMEM (100 μL/well) at various concentrations (6.25, 12.5, 25, 50, and 100 μM) were added to each well carefully. These cultured plates were incubated in humid atmosphere for another 24, 48, or 72 h. In total, 5 mg/mL MTT (10 μL/well) solution was added to every well of the 96-well plate. After 4-h incubation, DMSO (100 μL) was added to each well to dissolve the formazan. The data were obtained at 570/680 nm using an enzyme-linked immunosorbent assay (ELISA) reader (VARIOSKAN FLASH). The following formula was used to calculate the percentage viability of cells: viability (%) = (mean of absorbance value of treatment group/mean of absorbance value of control) × 100.

#### Cell imaging

Cultured HeLa cell dishes were carefully washed with PBS (pH 7.4) and then 10 μM **L** in serum-free DMEM was added to each cell culture dish. These dishes were stored at 37 °C for 1 h prior to imaging. For Cu^2+^ imaging, cells were again incubated for 30 min with Cu^2+^ at a concentration of 10, 20, or 30 μM in serum-free DMEM followed by imaging. For PPi imaging, abovementioned cells were further incubated with various concentrations of PPi (20 μM, 40 μM, or 60 μM) for 30 min prior to imaging. Later, these cells were split into two groups (with/without ALP inhibitor) for time course fluorescence imaging of ALP activity at 30, 60, and 120 min.

#### Fluorescence sensing of PPi in synovial fluids of arthritis patients

The synovial fluids of arthritis patients were collected from the Shanghai Ninth Hospital, Shanghai Jiao Tong University School of Medicine. The patients have given their consent for the procedure and the doctors have the respective permissions to draw synovial fluids. Two samples were from arthritis patients while one sample was obtained from a healthy person as control. Prior to the fluorescence sensing, the synovial fluid samples were centrifuged with an ultrafiltration device (molecular weight cutoff 50 kDa; Millipore Amicon Ultra) at 6000 rpm for 20 min. Then, these samples were diluted with HEPES buffer (pH 7.4, 10 mM) in order to be consistent with the linear range of our method. For the fluorescent sensing of PPi in the synovial fluids, 10 μL diluted synovial fluid was added to the solution containing 5 μM **L** and 5 μM Cu^2+^ in HEPES buffer (pH 7.4, 10 mM) and incubated for 30 min.

#### Density functional theory calculations

To better evaluate the nature of coordination between **L** and Cu^2+^, the optimized structures of **L** and its corresponding Cu^2+^ complex have been calculated. All calculations were carried out by density functional theory (DFT) formalism implemented in Gaussian 09 quantum chemistry software package. Time-dependent DFT (TDDFT) calculation at the hybrid function density functional theory B3LYP level was carried out to find the excited states; molecular orbitals were built with the GaussView package.

### Syntheses and characterizations of **L** and **L**-Cu^2+^

The study was started with the synthesis of **L**. Briefly, 1,8-diaminonaphthalene in ethanol was added dropwise to an equivalent 4-methyl benzaldehyde and the colorless solution turned to light yellowish rapidly. Then, the mixture was heated to reflux for 2 h (ESM Scheme S1). The pure light yellowish solid product **L** was obtained and characterized after purification (ESM Figs. S1–S3). After synthesis, we first investigated the spectroscopic properties of **L**. The excitation spectrum of **L** in HEPES buffer (10 mM, 4% DMSO) was recorded (ESM Fig. S4). Further on, the absorption spectrum was observed at various pH values ranging from 5 to 9 in HEPES buffer (10 mM, 4% DMSO) at RT. UV-vis spectra of **L** at various pH values did not show obvious changes, suggesting that the chemical structure of **L** was stable to pH variations (ESM Fig. S5).

## Results and discussion

### In vitro detection of Cu^2+^

**Fig. 1:**
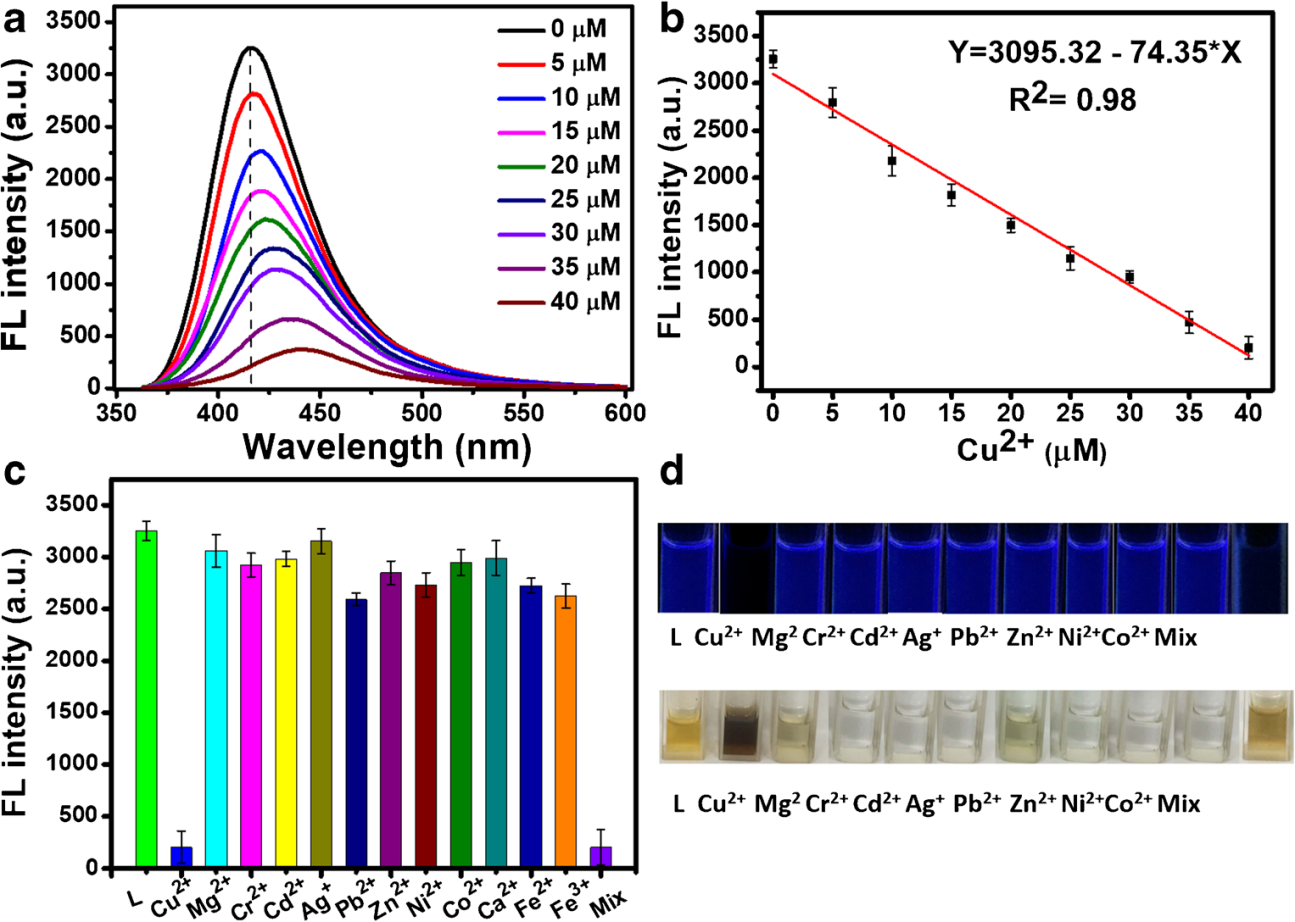
**a** Fluorescence spectra of 40 μM **L** in the presence of Cu^2+^ (0, 5, 10, 15, 20, 25, 30, 35, or 40 μM) in HEPES buffer (pH 7.4, 10 mM, 4% DMSO) at RT. *λ*_ex_ = 310 nm. **b** The fitted calibration line in the linear region of 0–40 μM Cu^2+^. **c** Fluorescence emissions of Cu^2+^ at RT were measured with 40 μM **L** before and after the addition of 3 equiv. of various ions (Mg^2+^, Cr^2+^, Cd^2+^, Ag^+^, Pb^2+^, Zn^2+^, Ni^2+^, Co^2+^, Ca^2+^, Fe2+, and Fe^3+^ or mix) in HEPES buffer (pH 7.4, 10 mM). *λ*_ex_ = 310 nm, *λ*_em_ = 415 nm. **d** Fluorescence responses of **L** to various ions under a UV lamp (top row) and photographs of **L** in presence of different metals (bottom row). These experiments were performed in triplicate; error bar represents standard deviation

After confirming the fluorescence property of **L**, we tested its capability for Cu^2+^ detection. An equivalent amount of Cu^2+^ was added into the abovementioned **L** solution. We observed a sharp decrease of the fluorescence (ESM Fig. S6A), probably due to entrapment of Cu^2+^ in **L** to form **L**-Cu^2+^ complex. Photographs showed that the light yellowish solution of **L** changed to a blackish green one after Cu^2+^ addition (ESM Fig. S6B). In detail, with the gradual addition of Cu^2+^ (from 0 to 40 μM), we observed a continuous decrease of the fluorescence peak of 40 μM **L**, accompanied by a 23-nm redshift from 415 to 438 nm (Fig. 1a). The FI at 415 nm and Cu^2+^ concentration showed a linear relation (*Y* = 3095.32 − 74.35*X, *R*^2^ = 0.98) over the range of 0–40 μM and the limit of detection (LOD) of Cu^2+^ was measured to be 2.60 μM according to the 3σ method (Fig. 1b), which is comparable with those of recently reported fluorescence probes for Cu^2+^ detection (Tab. S1). When the Cu^2+^ concentration exceeded 40 μM (**L**:Cu^2+^ = 1:1), no further decrease of the FI was observed, suggesting the binding stoichiometry between **L** and Cu^2+^ is 1:1. Electrospray ionization mass spectrum (ESI/MS) of **L** after the addition of 1 equiv. Cu^2+^ showed the dominant ionic peak in the spectrum has a *m*/*z* value of 393.08, which corresponds to [**L**-Cu^2+^] (ESM Fig. S7). Job’s plot analysis also indicated that the binding stoichiometry between **L** and Cu^2+^ is 1:1 (ESM Fig. S8). According to the reported method [54], the binding constant was calculated to be 1.263 × 10^5^ M^−1^. To further confirm the formation of the complexes, we directly synthesized CuCl_2_**L** complex by heating and stirring the dichloromethane/methanol solution of **L** with CuCl_2_ (1:1.2) at 50 °C for 3 h [55]. Transmission electron microscopy (TEM) images revealed the nanorod structure of CuCl_2_**L** with an average diameter of 65.3 ± 3.0 nm (ESM Fig. S9A). Energy-dispersive X-ray spectrometer (EDS) spectra of CuCl_2_**L** proved the existence of Cu in the complex (ESM Fig. 9B). The X-ray diffraction (XRD) analysis was employed to further identify CuCl_2_**L** complex formation. The XRD patterns of ligand, CuCl_2_, and **L**-Cu^2+^ complex were carefully analyzed, We observed that there were no specific peaks for ligand **L**, suggesting that the ligand is non-crystalline in nature. For CuCl_2_, an obvious clear peak was observed [56, 57]. However, for **L**-Cu^2+^ complex, we observed that some peaks were quite similar to CuCl_2_ peaks while some new peaks were also observed, suggesting that **L**-Cu^2+^ complex is successfully formed (ESM Fig. S10).

To evaluate the activity of newly designed probe, selectivity and interference are two important parameters. Especially for probes having biomedical applications, a selective response toward the target over various competing species is needed. Therefore, the selectivity study of **L** to Cu^2+^ over various metal ions, e.g., Co^2+^, Zn^2+^, Ni^2+^, Mg^2+^, Ag^+^, Pb^2+^, Cr^2+^, and Cd^2+^, was conducted. Moreover, interference from all above metal ions with the Cu^2+^ detection by **L** was investigated. As shown in Fig. 1c, **L** showed excellent selectivity and interference for Cu^2+^ detection among abovementioned metal ions. Besides, photographs showed clear color changes in cuvettes which contained Cu^2+^ alone or mixed with other metal ions; fluorescence of the samples under a UV lamp echoed that only the fluorescence from above two cuvettes was turned “Off” (Fig. 1d). These results confirmed the excellent selectivity and interference of **L** for Cu^2+^ detection in vitro*.*

### Computational study

**Fig. 2:**
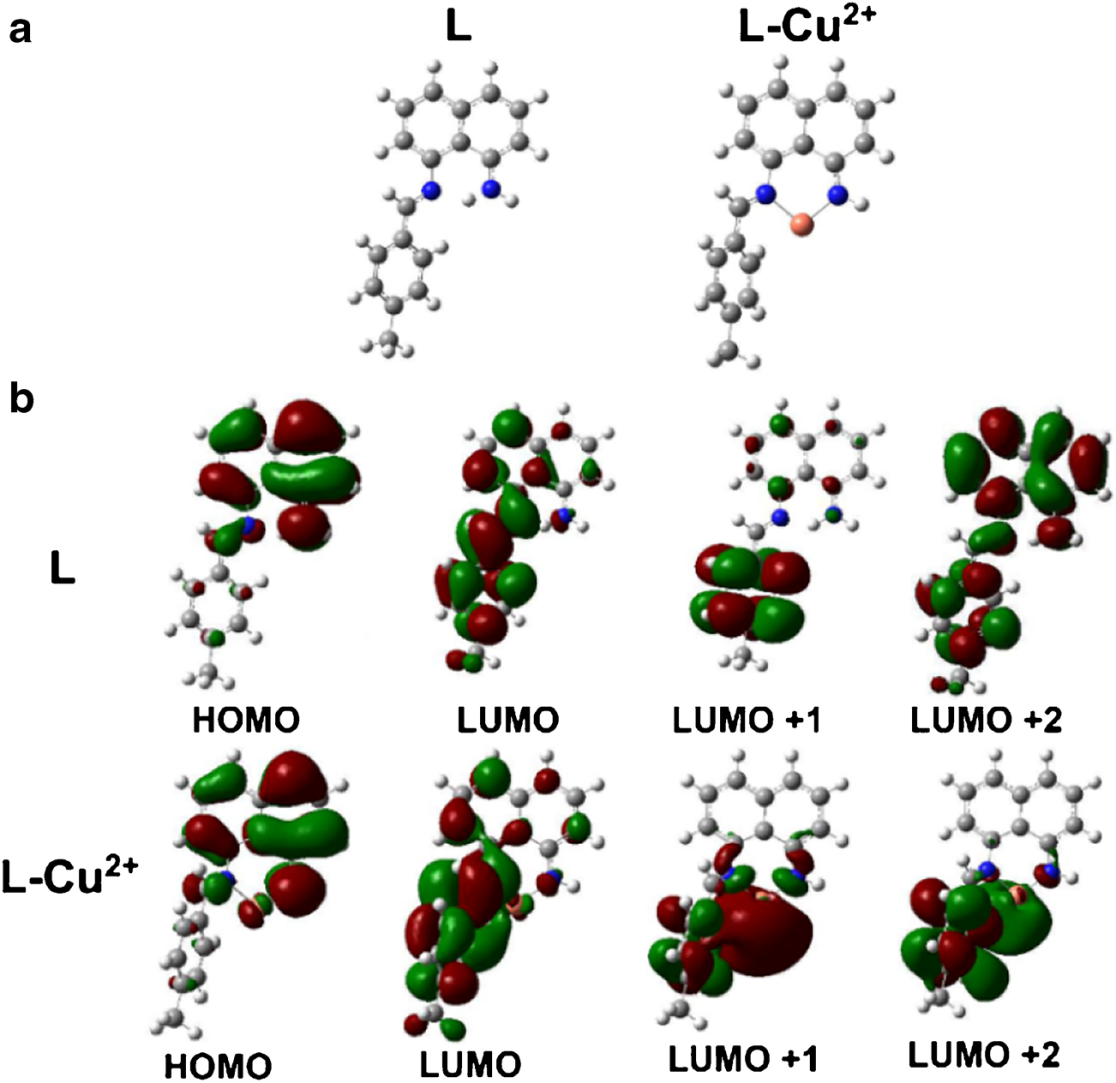
**a** Optimized structures of **L** (left side) and **L**-Cu^2+^ (right side). **b** B3LYP/6-31G*-calculated molecular orbitals of **L** (top row) and **L**-Cu2+ (bottom row)

To elaborate the mechanism of fluorescence quenching by the energy and/or charge transfer model, we carried out the computational calculations (Fig. 2). The optimized structures of **L** and **L**-Cu^2+^ were calculated at ground state (Fig. 2a). Time-dependent DFT (TDDFT) calculations at the hybrid DFT B3LYP level were carried out to find out the excited states. From this calculation, we noticed that the fluorescence quenching by Cu^2+^ could be rationalized in terms of the occupancy of the frontier orbitals. Figure 2 b shows the calculated frontier molecular orbitals (HOMO, LUMO, LUMO+1, LUMO+2) of **L** and **L**-Cu^2+^. These HOMO, LUMO, and LUMO+1 of both **L** and **L**-Cu^2+^ showed similar electron density distribution. The LUMO+2 of **L** showed equally distributed density over the whole structure while that of **L**-Cu^2+^ showed the electron density particularly over methylbenzene ring and Cu^2+^ ion, suggesting that the electron was transferred from the diaminonaphthalene ring to Cu^2+^ ion. This ligand to metal charge transfer (LMCT) mechanism caused the fluorescence quenching of **L** upon addition of Cu^2+^ [58].

### In vitro detection of PPi

**Fig. 3:**
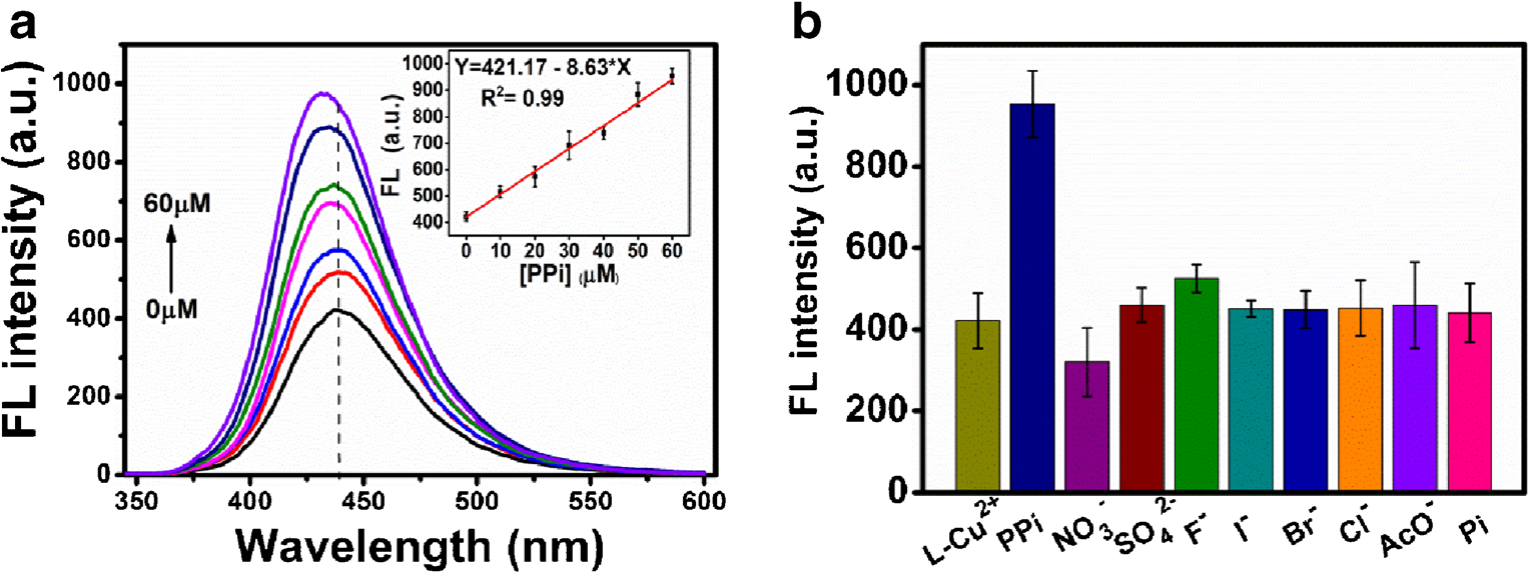
**a** Fluorescence spectra of 40 μM **L**-Cu^2+^ in the presence of PPi (0, 10, 20, 30, 40, 50, or 60 μM) in HEPES buffer (pH 7.4, 10 mM, 4% DMSO) at RT (*λ*_ex_ = 310 nm). Inset: the fitted calibration line in the linear region of 0–60 μM PPi. **b** FL of 40 μM **L**-Cu^2+^ complex at 438 nm with/without the addition of 5 equiv. of various common anions

As mentioned above, the binding affinity between Cu^2+^ and PPi is larger than that between **L** and Cu^2+^ [59]. Thus, addition of PPi to above **L**-Cu^2+^ solution will dissociate the **L**-Cu^2+^ complex to form new PPi-Cu^2+^ complex and turns the **L** fluorescence “On” again for PPi detection. As shown in Fig. 3a, with the increase of PPi concentration, fluorescence emission of the **L**-Cu^2+^ complex at 438 nm gradually increased accompanied by a 9-nm blueshift from 438 to 430 nm. Thus, a calibration curve of the FI of the **L**-Cu^2+^ complex at 438 nm for the determination of PPi in aqueous buffer was obtained (inset of Fig. 2a). It showed a linear relationship between the FI and PPi concentrations (*Y* = 421.17 − 8.63*X, *R*^2^ = 0.99) over the range of 0–60 μM. The LOD of PPi in this assay was calculated to be 0.31 μM (*S/N* = 3), which is comparable with those of recently reported fluorescence probes for PPi detection (Tab. S2). Moreover, the selectivity of **L**-Cu^2+^ complex to PPi among different common anions (NO_3_^−^, SO_4_^2−^, F^−^, I^−^, Br^−^, Cl^−^, and AcO^−^ in this work) was carefully studied. As shown in Fig. 3b, among the tested anions, only PPi induced a turned “On” fluorescence, suggesting excellent selectivity of the **L**-Cu^2+^ complex for PPi detection.

### Detecting pyrophosphate anion in synovial fluid samples

**Fig. 4:**
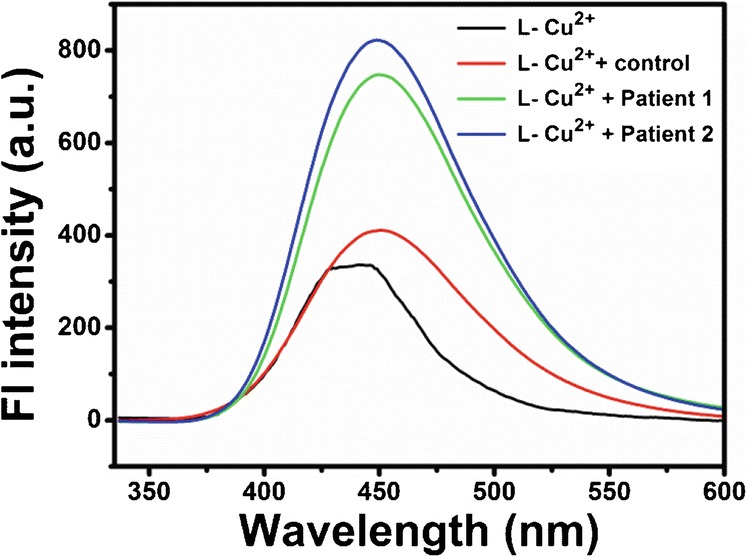
The fluorescence spectra of the reaction system carried out in synovial fluid from **L**-Cu^2+^ complex, healthy control, and two arthritis patients

The potential importance of our proposed design is its ability to operate in biological samples. To further demonstrate the potential application of our method in clinical samples, we detected PPi in synovial fluids from arthritis patients and healthy controls. As shown in Fig. 4, when **L**-Cu^2+^ complex was added to the synovial fluid of healthy control, we observed a 1.2-fold increase of FI. Compare with the healthy control, the Fl of **L**-Cu^2+^ complex with two synovial fluid samples from arthritis patients could further increase ∼ 1.4 and ∼ 1.8-fold, suggesting the elevated level of PPi in synovial fluids from arthritis patients. These results promote our findings that **L**-Cu^2+^ complex is quite suitable to apply for PPi detection in arthritis patients due to strong binding interaction between Cu^2+^ and PPi. Synovial fluid is a very complex medium and the redshift might be attributed to adsorption of medium compounds to the molecule or interaction with medium compounds.

### In vitro detection of ALP

**Fig. 5:**
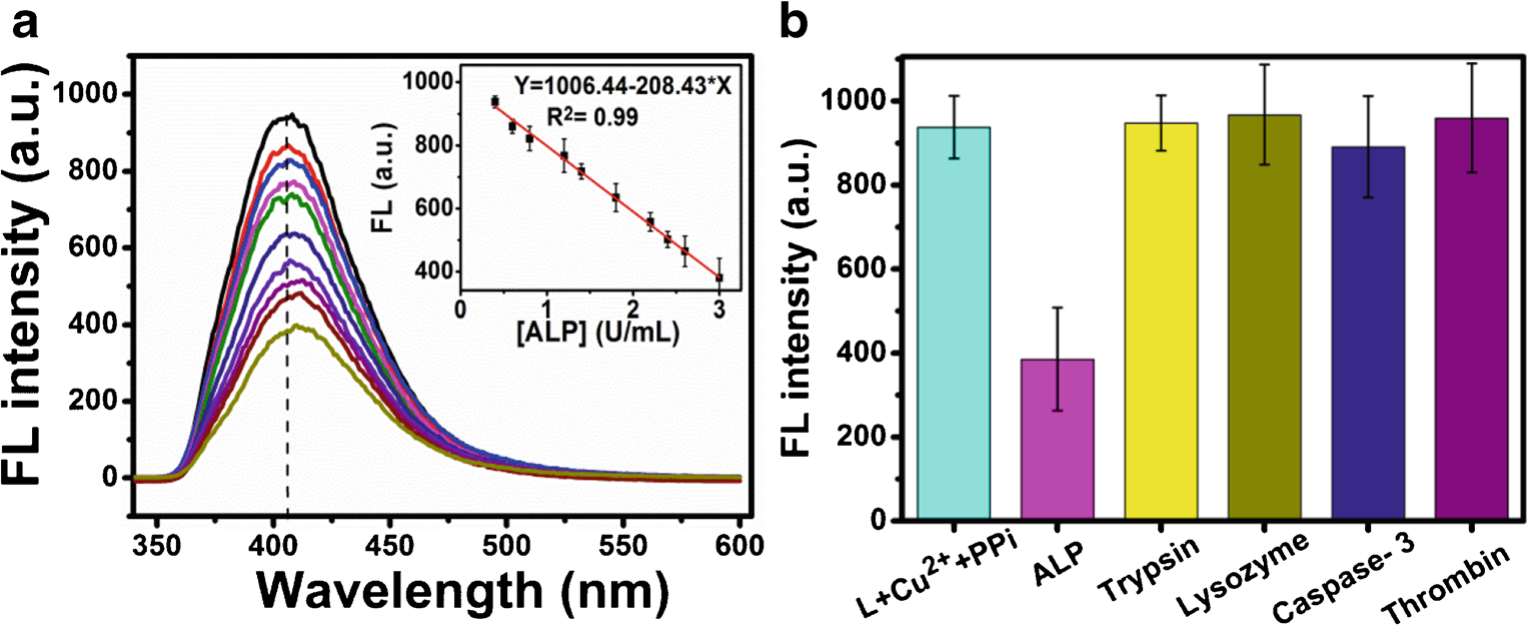
**a** Fluorescence spectra of 40 μM **L**-Cu^2+^ + 60 μM PPi in the presence of ALP at different concentrations (0.4, 0.6, 0.8, 1.2, 1.4, 1.8, 2.2, 2.4, 2.6, or 3.0 U/mL) in HEPES buffer (pH 7.4, 10 mM, 4% DMSO) at RT. *λ*_ex_ = 310 nm. Inset: the fitted calibration line in the linear region of 0.4–3.0 U/mL ALP. **b** FL of 40 μM **L**-Cu^2+^ + 60 μM PPi solution at 406 nm in the presence of ALP (3 U/mL), trypsin (50 U/mL), lysozyme (10 mg/ mL), caspase-3 (50 U/mL), and thrombin (50 μM)

Considering that ALP is able to dephosphorylate the PPi in the PPi-Cu^2+^ complex to free Cu^2+^ to re-chelate with **L**, we used the abovementioned mixture solution for quantitative detection of ALP activity with fluorescence “Turn-Off.” Following the formation of Cu^2+^-PPi, various concentrations of ALP (0.4 to 3.0 U/mL) were added and the fluorescence spectra were recorded after 30-min incubation at RT. As shown in Fig. 5a, with the increase of ALP concentration, Fl of the mixture solution at 406 nm gradually decreased. By correlating the FI the mixture solution at 406 nm with the concentration of ALP, we obtained a calibration curve for the determination of ALP in aqueous buffer (inset in Fig. 5a). A linear relationship between the Fl at 406 nm and ALP concentration (*Y* = 1006.44 − 208.43*X, *R*^2^ = 0.99) was obtained (0.4–3.0 U/mL), with LOD value 0.05 U/mL (*S*/*N* = 3) of ALP in this assay, which is comparable with those of recently reported fluorescence probes for ALP detection (Tab. S3). Selectivity study of as-formed Cu^2+^-PPi complex in the solution to ALP over various enzymes (trypsin, lysozyme, caspase-3, and thrombin in this work) indicated that the mixture solution is selective for ALP detection in vitro with fluorescence “Turn-Off” property (Fig. 5b).

### Sequential imaging of Cu^2+^ and PPi in living cells

**Fig. 6:**
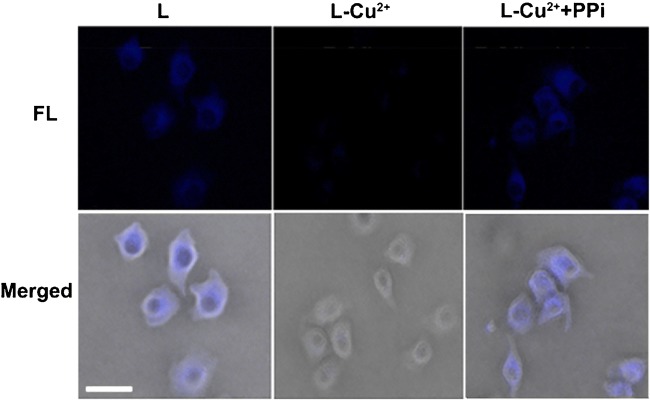
Fluorescence images (top row) and merged (DIC + FL) images (bottom row) of HeLa cells incubated with 10 μM **L** in serum-free DMEM for 1 h at 37 °C, washed with PBS (left column), added with 30 μM Cu^2+^ for 30 min (middle column) and then 60 μM PPi for another 30 min (right column) in serum-free DMEM at 37 °C prior to imaging. Scale bar: 20 μm

Before cell imaging studies, cytotoxicity of **L** was investigated on living HeLa cells. MTT results indicated that, up to 100 μM and 72 h, **L** did not impose obvious cytotoxicity on the cells, suggesting 10 μM **L** was safe for live cell imaging (ESM Fig. S11). HeLa cells were incubated with 10 μM **L** at 37 °C for 1 h in serum-free DMEM and then washed with PBS for three times prior to imaging. Bright blue fluorescence from cell cytoplasm was observed, suggesting our probe **L** is cell permeable (left column of Fig. 6). Then, the cells were incubated with Cu^2+^ (0, 10, 20, or 30 μM) at 37 °C for 30 min. Fluorescence cell imaging showed that the Fl gradually decreased with the increase of Cu^2+^ concentration (ESM Fig. S12) and was effectively quenched at Cu^2+^ concentration of 30 μM (ESM Fig. S12 and the middle column of Fig. 6). The average FL of HeLa cells in ESM Fig. S12 was measured by using ImageJ and is summarized in ESM Fig. S13. The Fl of **L** in Hela cells had a 7.4-fold decrease by 30 μM Cu^2+^. After that, the 30 μM Cu^2+^-treated cells were incubated with various concentrations of PPi at 0, 20, 40, or 60 μM for another 30 min prior to imaging. Clearly, we found that cell fluorescence gradually turned “On” again (ESM Fig. S14) and reached its plataeu at 60 μM PPi concentration (ESM Fig. S14 and the right column of Fig. 6). The average FL of HeLa cells in ESM Fig. S14 was measured by using ImageJ and is summarized in ESM Fig. S15. The Fl of **L**-Cu^2+^ in Hela cells had a 7.6-fold increase by 60 μM PPi. With the “Off” and “On” fluorescence property of **L** after Cu^2+^ and PPi additions, we successfully applied **L** for imaging Cu^2+^ and PPi in living cells.

### Imaging of ALP in living cells

**Fig. 7:**
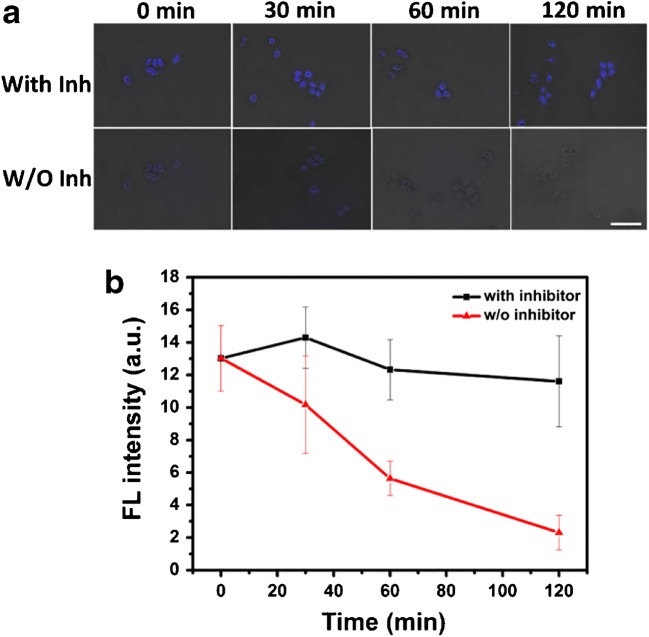
**a** Fluorescence imaging of the Cu^2+^- and PPi-pretreated HeLa cells incubated with (top row) or without (bottom row) phosphatase inhibitor complex II and imaged at different time points (0, 30, 60, and 120 min). Scale bar: 50 μm. **b** FL plots of the cells in A. These experiments were performed in triplicate. Error bars represent standard deviations

In order to apply our probe for imaging ALP activity in living HeLa cells, we split above PPi-treated cells into two groups: one group was treated with ALP inhibitor prior to imaging while the other was without any pretreatment. In detail, the 30 μM Cu^2+^-treated and 60 μM PPi-treated cells were incubated with/without phosphatase inhibitor complex II (10 μL ALP inhibitor complex II in 1 mL culture medium) and fluorescence images were taken at 30, 60, and 120 min at 37 °C after inhibitor addition. The results indicated that the Fl inside the inhibitor-treated cells maintained over the observation time (top row in Fig. 7a and black line in Fig. 7b). However, Fl inside the cells without any pretreatment decreased over time (bottom row in Fig. 7a and red line in Fig. 7b). Above results suggested that the decreased FI in cells without treatment was induced by intracellular ALP which dissociated the PPi-Cu^2+^ complex to free Cu^2+^ to re-chelate with **L**, turning the fluorescence “Off.” With this property, our probe **L** could be employed for imaging intracellular ALP activity in living cells pretreated with Cu^2+^ and PPi.

## Conclusion

In conclusion, we rationally designed a new fluorescent probe (E)-8-((4-methylbenzylidene)amino)napthalen-1-amine (**L**) for sequential and selective detections of Cu^2+^, PPi, and ALP in vitro and in living cells with fluorescence “Off,” “On,” and “Off” signals, respectively. The working mechanisms underlying these processes are as follows: (1) chelation between Cu^2+^ and **L** yields the **L**-Cu^2+^ complex, accompanied by fluorescence “Off” due to the LMCT effect; (2) due to the stronger binding affinity of PPi-Cu^2+^ than **L**-Cu^2+^, addition of PPi to the **L**-Cu^2+^ solution dissociates the **L**-Cu^2+^ complex and turns the fluorescence “On”; (3) further added ALP hydrolyzes PPi in the PPi-Cu^2+^ complex, freeing Cu^2+^ to re-chelate with **L** and turn the fluorescence “Off.” In physiological solutions, **L** was successfully applied for selective detections of Cu^2+^, PPi, and ALP in vitro with LODs of 2.60 μM, 0.31 μM, and 0.05 U/mL, respectively. Moreover, with its property of fluorescence switch, **L** was successfully applied to image Cu^2+^, PPi, and ALP in living HeLa cells. Considering the obvious correlations among Cu^2+^, PPi (product of ATP), and ALP in Wilson’s disease, we envision that our fluorescent probe **L** could be applied to in vitro diagnose WD in the near future.

## Electronic supplementary material

ESM 1 (DOCX 2.48 MB)
