# Supplementary material for: Smart probe for simultaneous detection of copper ion, pyrophosphate, and alkaline phosphatase in vitro and in clinical samples
Source: Anal Bioanal Chem. 2019 Aug 2;411(24):6475–85. doi: 10.1007/s00216-019-02027-2 (PMC6718369; doi:10.1007/s00216-019-02027-2)
Supplement: Supplementary file 1 — (DOCX 2.48 MB) [file 216_2019_2027_MOESM1_ESM.docx]

**Analytical and Bioanalytical Chemistry**

**Electronic Supplementary Material**

Smart probe for simultaneous detection of copper ion, pyrophosphate, and alkaline phosphatase in vitro and in clinical samples

Sonia Kiran, Renuka Khatik, Romana Schirhagl

**Contents**

**1**. **Syntheses and Characterizations of L**

**2. Supporting Figures**

**3. Supporting Tables**

**4. References**

**1. Syntheses and Characterizations of L**

1,8-diaminonaphthalene (2.3 g, 15 mmol) was dissolved in 15 mL ethanol in a 50 mL round bottom flask. 1.8 g of 4-methyl benzaldehyde (15 mmol) was dissolved in ethanol (15 mL) and added to the same flask dropwise. The solution turned yellowish white rapidly and was heated to reflux for 2 hours. The reaction mixture was then cooled to room temperature for slow evaporation. The yellowish white solid product was filtered and washed with cold ethanol and dried under vacuum (scheme 1). Yield, 75%. ^1^H NMR (400 MHz, DMSO-*d*_6_) δ (ppm) : 7.48 (d, *J* = 7.7 Hz, 2 H), 7.23 (d, *J* = 7.7 Hz, 2 H), 7.14 (t, *J* = 7.8 Hz, 2 H), 6.97 (d, *J* = 8.2 Hz, 2 H), 6.69 (s, 2 H), 6.48 (d, *J* = 7.4 Hz, 2 H), 5.31 (s, 1 H), 2.33 (s, 3 H) (Fig. S1). ^13^C NMR (100 MHz, DMSO-*d*_6_) δ (ppm) :143.65, 139.33, 138.23, 134.95, 129.19, 128.30, 127.44, 115.70, 112.99, 105.21, 66.71, 21.29 (Fig. S2). MS: calculated for **L** [(M+H)^+^]: 261.14, obsvd. ESI-MS: m/z 261.18 (Fig. S3).

**Scheme 1** Synthetic route for **L**


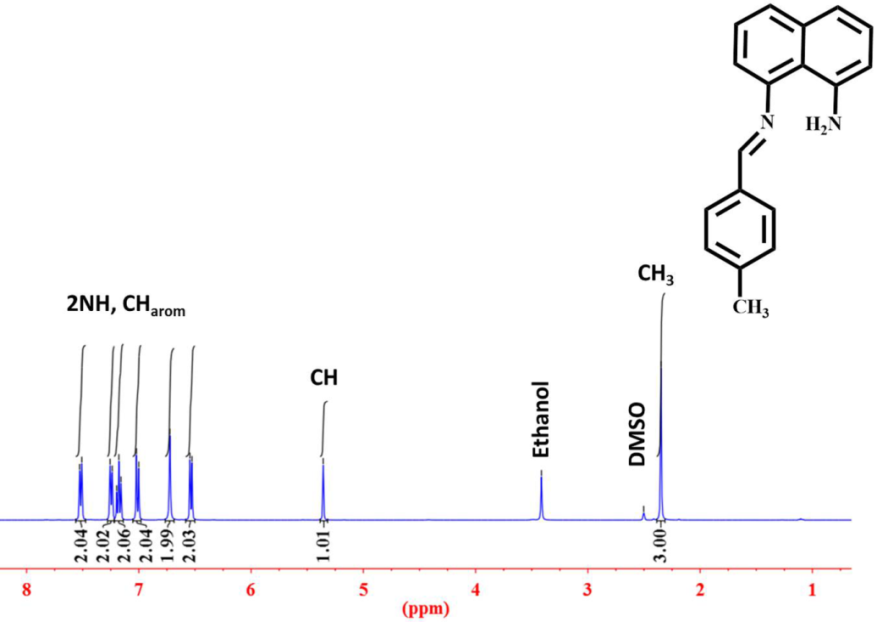


**Fig. S1** ^1^H NMR spectrum of **L** in d_6_-DMSO


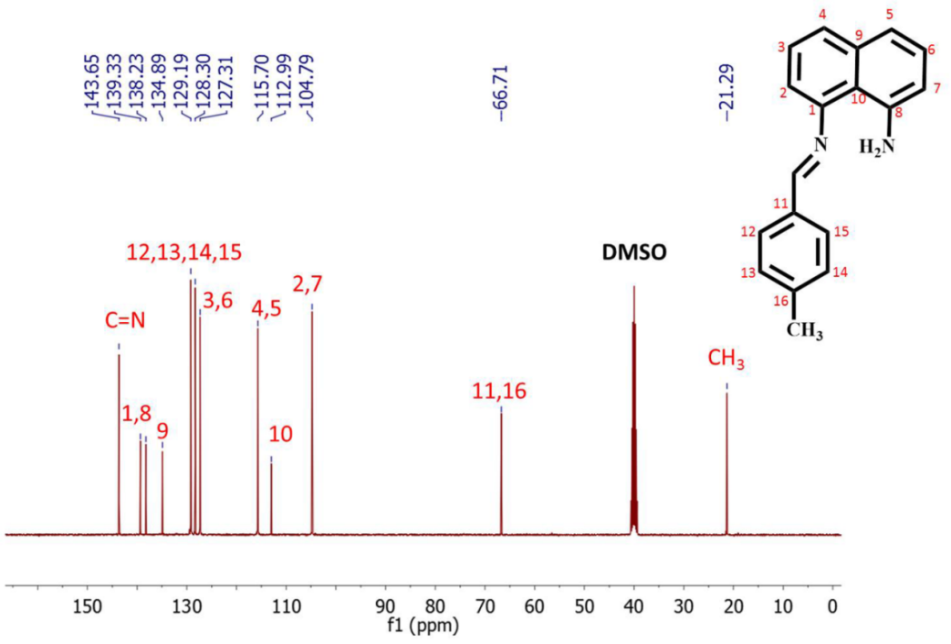


**Fig. S2** ^13^C NMR spectrum of **L** in *d*_6_-DMSO


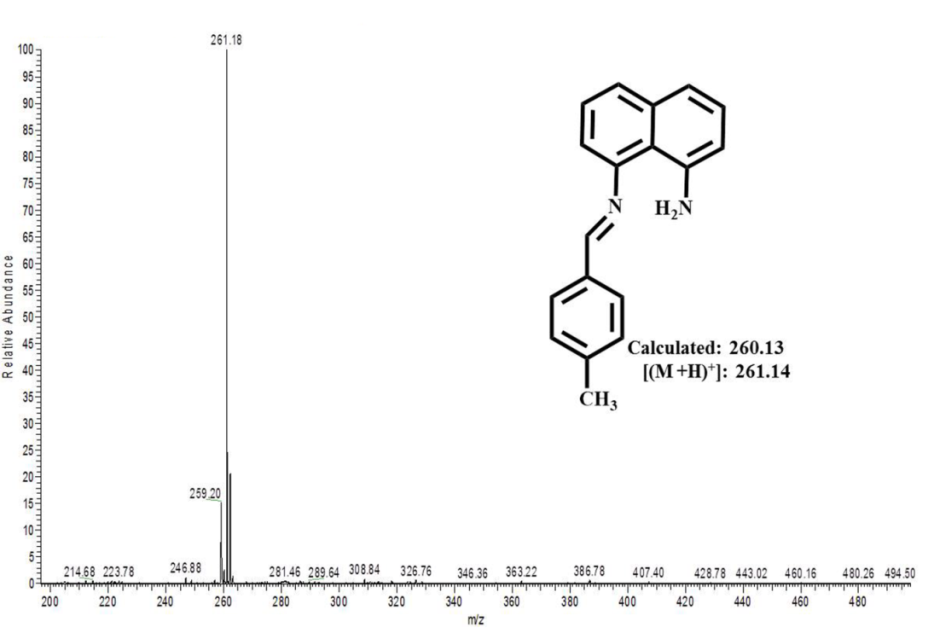


**Fig. S3** ESI/MS spectrum of **L**

**2. Supporting Figures**


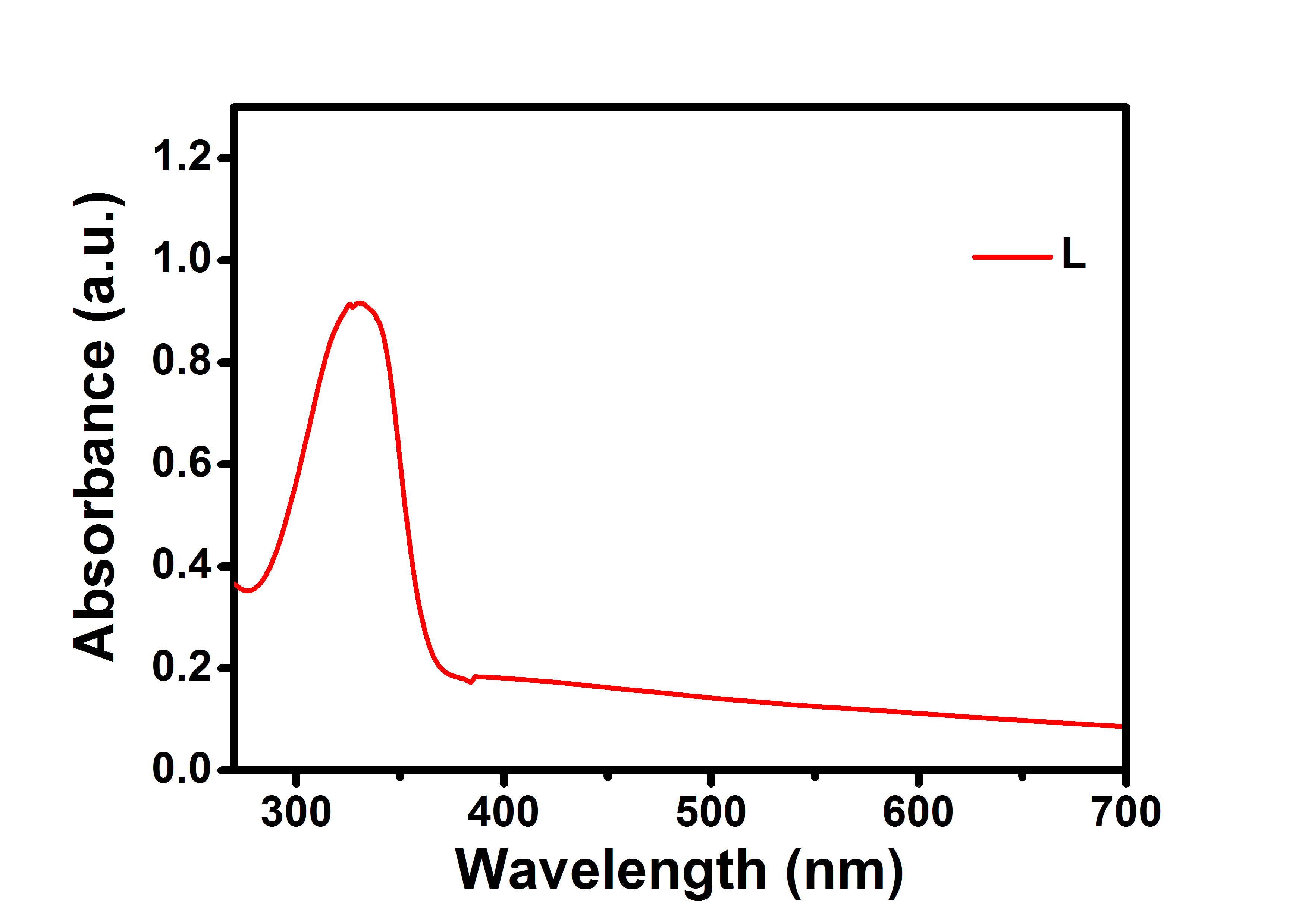


**Fig. S4** Excitation spectrum of L in HEPES buffer (10 mM, 4% DMSO)


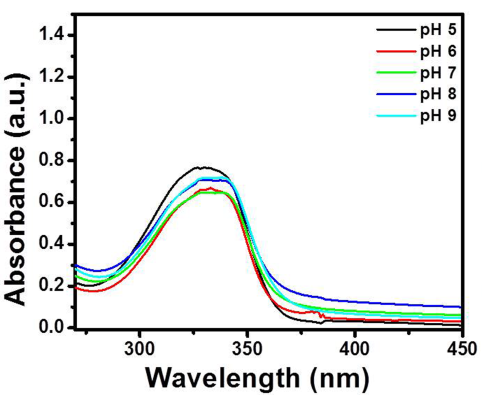


**Fig. S5** UV−vis spectra of **L** in HEPES buffer (10 mM, 4% DMSO) at different pH values (5-9)


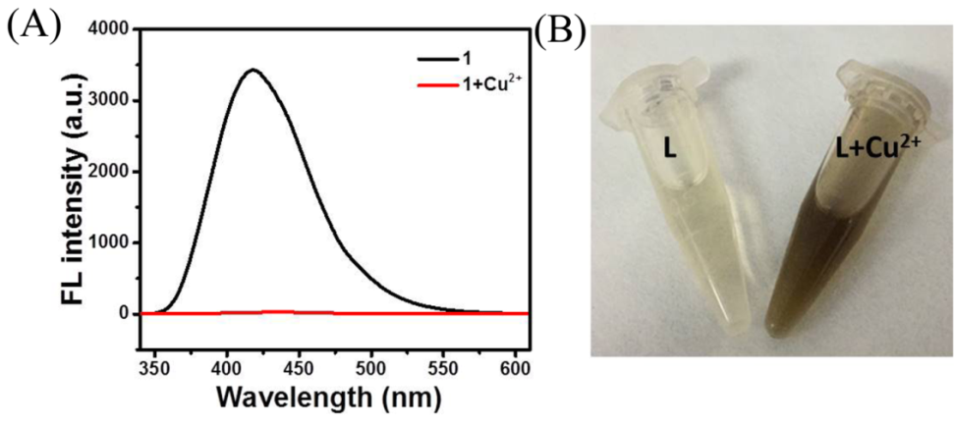


**Fig. S6** (A) Fluorescence spectra of **L** (black) and **L**-Cu^2+^ (red) in HEPES buffer at pH 7.4. Excitation wavelength: 310 nm. (B) Visual detection of the formation of **L**-Cu^2+^ complex


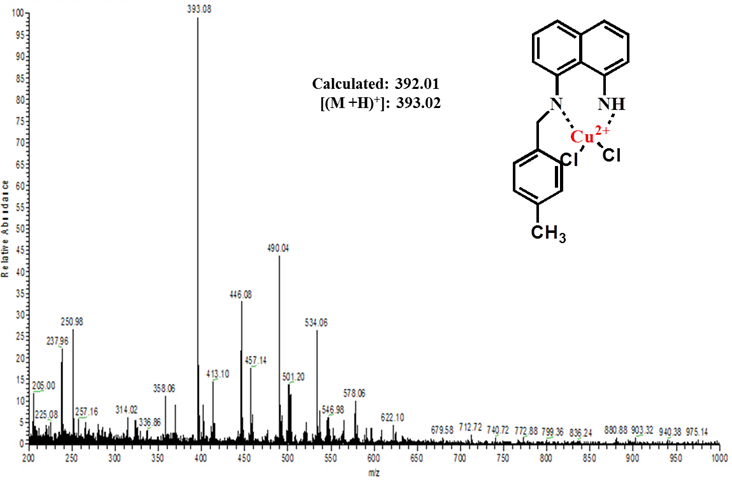


**Fig. S7** ESI/MS spectrum of **L**-Cu^2+^


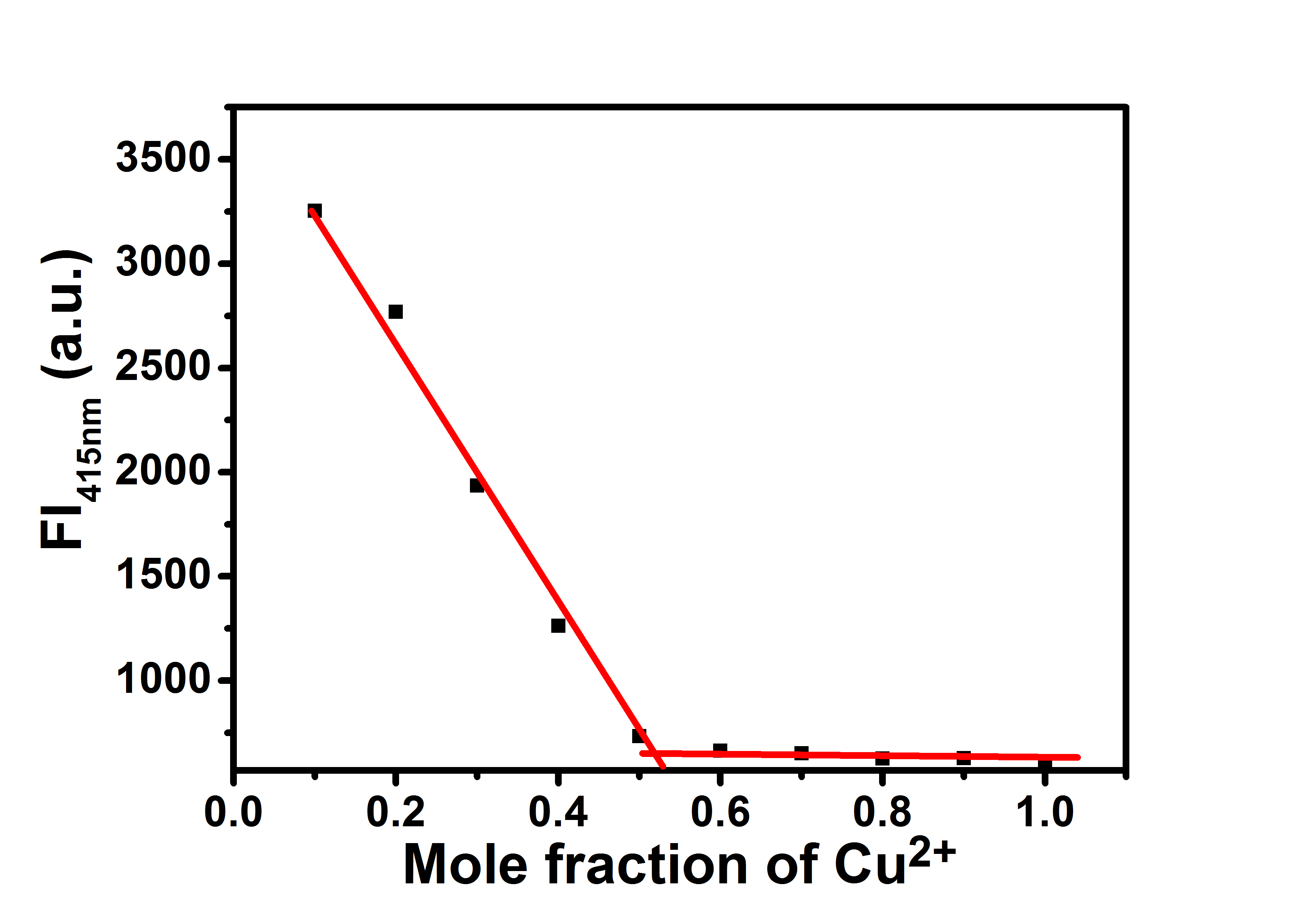


**Fig. S8** Job’s plot analysis between **L** with Cu^2+^ (**L**+ Cu^2+^ = 40 μM) in HEPES buffer (10 mM, pH 7.4, 4% DMSO) at RT. Excitation and emission was at 310 and 415 nm, respectively

*
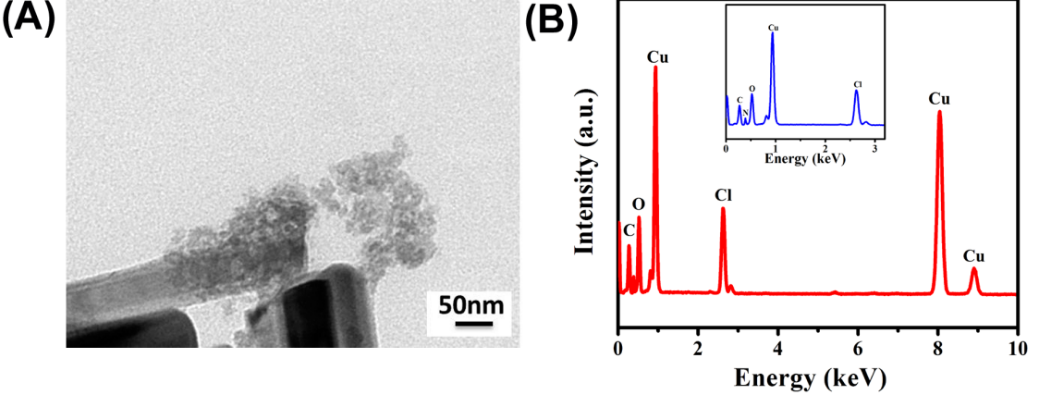
*

**Fig. S9** (A) TEM image and (B) EDS spectrum of CuCl_2_**L**

**Fig. S10** X-ray diffraction (XRD) patterns of **L**, CuCl_2_, and **L**-Cu^2+^ complex

**Fig. S11** MTT assay of probe **L** on HeLa cells. The cells were incubated with different concentrations (6.25, 12.5, 25, 50, and 100 µM) of **L** for 1, 2, and 3 days respectively. The experiments were performed in triplicate. Error bars represent standard deviations


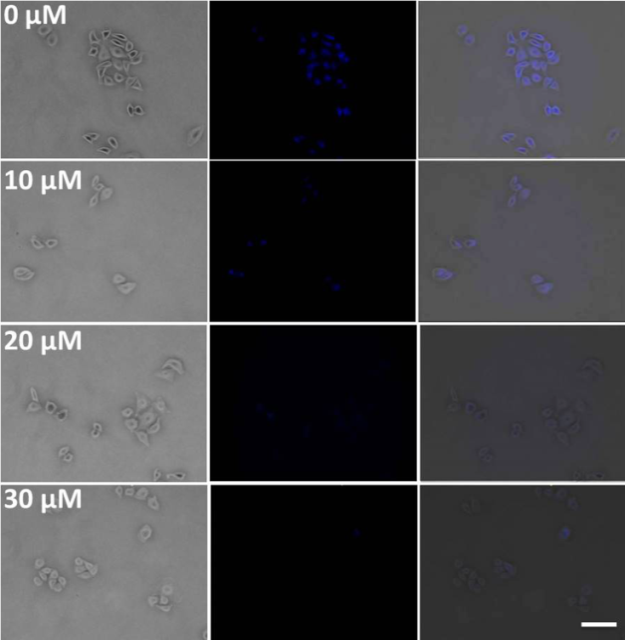


**Fig. S12** Differential interference contrast (DIC) images (left column), fluorescence images (middle column,DAPI channel) and overlay (right column) of HeLa cells incubated with 10 μM **L** in serum-free DMEM for 1 hour at 37 °C, washed with PBS for three times, then incubated with 0, 10, 20, or 30 μM of Cu^2+^ in serum-free DMEM for 30 minutes at 37 °C prior to imaging, respectively. Scale bar: 50 μm

**Fig. S13** The average FI of HeLa cells fluorescence in Fig. S13. The experiments were performed in triplicate. Error bars represent standard deviations


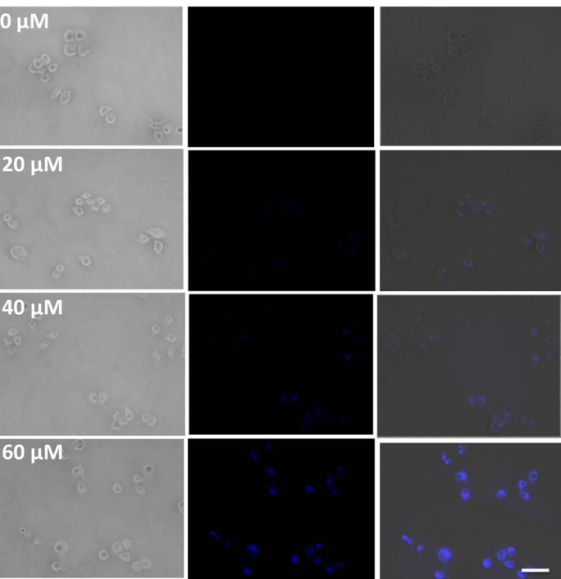


**Fig. S14** Differential interference contrast (DIC) images (left column), fluorescence images (middle column, DAPI channel) and overlay (right column) of HeLa cells incubated with 10 μM **L** in serum-free medium for 1 hour at 37 °C, washed with PBS for three times, incubated with 30 μM of Cu^2+^ in serum-free DMEM for 30 minutes at 37 °C, then incubated with 0, 20, 40, or 60 μM of PPi in serum-free DMEM for 30 minutes at 37 °C prior to imaging, respectively. Scale bar: 50 μm

**Fig. S15** The average FI of HeLa cells fluorescence in Fig. S15. The experiments were performed in triplicate. Error bars represent standard deviations

**3. Supporting Tables**

**Table S1** Summary of detection parameters of **L** and recently reported Cu^2+^ probes

| **Limit of Detection** | **Linear detection range** | **Buffer** |
| --- | --- | --- |
| 2.60 µM | 0-40 μM | HEPES buffer (pH 7.4, 10 mM, 4% DMSO) |
| 0.0416 μM^1^ | 0-100 μM | Tris-HCl buffer (5 mM, pH 7). |
| 0.30 µM^2^ | 1.0-160 µM | Tris-HCl (5 mM, pH 7.2, 0.1 M KCl) |
| 0.25 μM^3^ | 0.25-20 μM | 1/1 v/v; HEPES 100 mM; pH 7.0 |
| 10 nM^4^ | 1-14 μM | 50% (v/v) H_2_O/CH_3_CN buffered by 10 mM HEPES. |
| 2.73 μM^5^ | 0-25 μM | 5 mM HEPES, pH 7.0 |
| 3 μM^6^ | 0-250 µM | Tris-HCl buffer (CH_3_CN–water, 9 : 1; pH 7.2). |
| 0.1129 μM^7^ | 0-30 µM | Tris-HCl, pH 6.8 |

**Table S2** Summary of detection parameters of **L**-Cu^2+^ and recently reported PPi probes

| **Limit of Detection** | **Linear detection range** | **Buffer** |
| --- | --- | --- |
| 0.31 μM | 0-60 μM | HEPES buffer (pH 7.4,10 mM, 4% DMSO |
| 3.39 μM^8^ | 0-13 μM | CH_3_CN/10 mM HEPES=1:1, pH 7.4 |
| 2.02 μM^9^ | - | 10 mM MOPS, pH = 7.0 |
| 2 μM^10^ | 0-30 μM | 10 mM Tris–HCl, pH 7.4 |
| 1.5 μM^11^ | 5.0 μM-1.0 μM | CH_3_CN/20 mM HEPES=5: 95,  pH 7.4) |
| 9.72 nM^12^ | 0.4 μM-3.2 μM | HEPES buffer (pH = 7.4, 10 mM in H_2_O/DMSO, 7/3, v/v) |

**Table S3** Summary of detection parameters of **L**-Cu^2+^-PPi and recently reported ALP probes

| **Limit of Detection** | **Linear detection range** | **Buffer** |
| --- | --- | --- |
| 0.05 U/mL | 0-3 U/mL | pH 7.4, 10 mM, 4% DMSO |
| 1.09 U/L^13^ | 0-1.0 U/mL | 10 mM Tris-HCl buffer (pH 7.4) |
| 0.38 U/L^14^ | 0-50 U/mL | Tris–HCl buffered (pH 8.0, 10mM) |
| 0.1 U/mL^15^ | 0-20 U/mL | Tris−HCl buffer (pH 8.0, 10 mM) |
| 0.15 U/L^16^ | 0-200 mU/mL | Tris-HCl buffer solution (pH = 9.2 |
| 3.8 U/L^17^ | 10-500 U/L | Tris–HCl buffer solution (pH = 7.4) |
| 0.06 U/mL^18^ | 0-2.8 U/mL | Tris-HCl (50 mM, pH 8.0) |

**4. References**

[1] S. H. Mashraqui, K. Poonia, R. Betkar, M. Chandiramani, A novel retro-reaction strategy toward designing a selective fluorescence Cu(II) chemodosimeter, Tetrahedron Lett. 51 (2010) 4336-4339.

[2] A. F. Li, H. He, Y. B. Ruan, Z. C. Wen, J. S. Zhao, Q. J. Jiang, Y. Jiang, Oxidative cyclization of N-acylhydrazones. Development of highly selective turn-on fluorescent chemodosimeters for Cu^2+^, Org. Biomol.Chem. 7 (2009) 193-200.

[3] D. Wang, Y. Shiraishi, T. Hirai, A BODIPY-based fluorescent chemodosimeter for Cu(II) driven by an oxidative dehydrogenation mechanism, Chem. Commun. 47 (2011) 2673-2675.

[4] Z. Q. Hu, X. M. Wang, Y. C. Feng, L. Ding, H. Y. Lu, Sulfonyl rhodamine hydrazide: A sensitive and selective chromogenic and fluorescent chemodosimeter for copper ion in aqueous media, Dyes Pigm. 88 (2011) 257-261.

[5] H. Wang, S. Wu, A pyrene-based highly selective turn-on fluorescent sensor for copper(II) ions and its application in living cell imaging, Sens. Actuator B-Chem. 181 (2013) 743-748.

[6] Y. Chen, C. Zhu, J. Cen, J. Li, W. He, Y. Jiao , Z. Guo, A reversible ratiometric sensor for intracellular Cu2+

imaging: metal coordination-altered FRET in a dual fluorophore hybrid, Chem. Commun. 49 (2009) 7632—7634.

[7] X. Zeng, C. Wu, L. Dong, L. Mu, S. Xue, Z. Tao, A new tripodal rhodamine B derivative as a highly selective and sensitive fluorescence chemosensor for copper(II), Sci. China Ser. B-Chem. 52 (2009) 523-528.

[8] S. Y. Jiao, K. Li, W. Zhang, Y. H. Liu, Z. Huang, X. Q. Yu, Cd(II)-terpyridine-based complex as a ratiometric fluorescent probe for pyrophosphate detection in solution and as an imaging agent in living cells, Dalton Trans. 44 (2015) 1358-1365.

[9] W. Zhu, X. Huang, Z. Guo, X. Wu, H. Yu, H. Tian, A novel NIR fluorescent turn-on sensor for the detection of pyrophosphate anion in complete water system, Chem. Commun. 48 (2012) 1784-1786.

[10] Y. Chen, W. Li, Y. Wang, X. Yang, J. Chen, Y. Jiang, C. Yu, Q. Lin, Cysteine-directed fluorescent gold nanoclusters for the sensing of pyrophosphate and alkaline phosphatase, J. Mater. Chem. C. 20 (2014) 4080-4085.

[11] J. F. Zhang, S. Kim, J. H. Han, S. J. Lee, T. Pradhan, Q. Y. Cao, S. J. Lee, C. Kang, J. S. Kim, Pyrophosphate-Selective Fluorescent Chemosensor Based on 1,8-Naphthalimide–DPA–Zn(II) Complex and Its Application for Cell Imaging, Org. Lett. 13 (2011) 5294-5297.

[12] D. Chao, S. Ni, Nanomolar pyrophosphate detection and nucleus staining in living cells with simple terpyridine–Zn(II) complexes, Sci. Rep. 6 (2016) 26477.

[13] H. Zhang, C. Xu, J. Liu, X. Li, L. Guo, X. Li, An enzyme-activatable probe with a self-immolative linker for rapid and sensitive alkaline phosphatase detection and cell imaging through a cascade reaction, Chem. Commun. 51 (2015) 7031-7034.

[14] X. Hou, Q. Yu, F. Zeng, J. Ye, S. J. Wu, A ratiometric fluorescent probe for in vivo tracking of alkaline phosphatase level variation resulting from drug-induced organ damage, J.Mater. Chem. B. 3 (2015) 1042-1048.

[15] F. Zheng, S. Guo, F. Zeng, J. Li, S. Wu, Ratiometric fluorescent probe for alkaline phosphatase based on betaine-modified polyethylenimine via excimer/monomer, Anal. Chem. 86 (2014) 9873−9879.

[16] Z. Song, R. T. K Kwok, E. Zhao, Z. He, Y. Hong, J. W. Y. Lam, B. Liu, B. Z. Tang, A Ratiometric Fluorescent Probe Based on ESIPT and AIE Processes for Alkaline Phosphatase Activity Assay and Visualization in Living Cells, ACS Appl. Mater. Interfaces 6 (2014) 17245-17254.

[17] Z. Lu, J. Wu, W. Liu, G. Zhang, P. Wang, A ratiometric fluorescent probe for quantification of alkaline phosphatase in living cells, RSC Adv. 6 (2016) 32046-32051.

[18] L. Dong, Q. Miao, Z Hai, Y. Yuan, G. Liang, Enzymatic Hydrogelation-Induced Fluorescence Turn-Off for Sensing Alkaline Phosphatase in Vitro and in Living Cells, Anal. Chem. 87 (2015) 6475−6478.
